# The construction of a new Clinical Quality of Life Scale (CLINQOL)

**DOI:** 10.1186/s40359-022-00912-7

**Published:** 2022-08-30

**Authors:** Patrick Jones, Peter Drummond

**Affiliations:** College of Science, Health, Engineering and Education (SHEE), Murdoch University, Perth, WA Australia

**Keywords:** Questionnaire, Quality of life, Well-being, Mindfulness, Assessment, Clinical Quality of Life Scale (CLINQOL)

## Abstract

Whilst the variables in quality of life and well-being can be separated into objective and subjective domains, there remains a dearth of multiple utility instruments that assess their impact upon quality of life. To address this, ten domains were identified in a preliminary exploratory literature search, and an exploratory review generated enough facets to represent each domain, with items developed to form a composite scale. A principal components analysis run on data collected from 210 participants produced seven factors: relationships, work, money, health, leisure, and life management, with the remaining four domains subsuming into a seventh composite subjective factor (mental state). Final items were collated into the new Clinical Quality of Life Scale (CLINQOL) and were tested against the Assessment of Quality of Life Instrument, the Personal Wellbeing Index–Adult, Positive and Negative Affect Scale, the Satisfaction with Life scale, and the Mindfulness Attention Awareness Scale. The CLINQOL demonstrated suitable reliability, with items within each category forming internally consistent subscales. The full scale score demonstrated satisfactory test–retest reliability and concurrent validity, correlating with all measures. Findings suggest that the CLINQOL captures critical clinical factors, and may be an acceptable instrument to assess quality of life and well-being.

**Supplementary Information:**

The online version contains supplementary material available at 10.1186/s40359-022-00912-7.

## Introduction

In view of the largely empirical nature of quality of life assessment, the need for valid and reliable psychometric measurements is critical. In early research on subjective well-being, assessment approaches often relied upon a single, self-report item to measure each construct. Used to obtain a global self-report of well-being, the early scales however generally possessed adequate psychometric properties, good internal consistency, moderate stability and appropriate sensitivity to changing life circumstances [1–3].

For example, one of the earliest measures of a component of well-being was the Affect Balance Scale [4]. This measures the level of balance between five positive affect items and five negative affect items. Similarly, the Delighted-Terrible Scale of Andrews and Withey [5] uses a single item with a 7-point response format. This item measures how an individual feels about life at the present time. Diener et al.’s [6] popular Satisfaction with Life Scale uses five items to address five different cognitive or judgmental processes by which people assess life satisfaction.

Whilst the items of the above scales generate reliable and well tested global assessments of affect and cognition in respect of quality of life, their specificity and simplicity carry its own limitations. For example, as there are many dimensions to quality of life measurement, these necessarily get missed by the scales’ brevity. This trend has been carried through in scale development for quality of life and represents a weakness that needs to be addressed in future measures.

A more wide-ranging measure that attempted to address this was the Quality of Life Inventory (QOLI) [7] which was developed to measure individuals' ratings on 17 dimensions considered relevant to life satisfaction. Frisch et al. [8] created two response categories, in the first, participants rated their satisfaction in each quality of life area and in the second participants rated this area's importance in their life. This second dimension enabled the derivation of a more customised weighted scale.

The QOLI reported internal consistencies ranging from 0.77 to 0.89 whilst test–retest reliability has been reported as ranging from 0.80 to 0.91 across 3 clinical and 3 nonclinical samples. Lyubomirsky and Lepper [9] developed a four item scale measuring global subjective happiness. Two items ask respondents to characterise themselves on absolute rating and ratings relative to peers. The other two items are descriptions of happy and unhappy people which participants rate themselves against. Internal consistency ranges from 0.79 to 0.94 with a median of 0.86 whereas test–retest reliability ranges from 0.55 to 0.90.

In terms of specific characteristics, the Schedule for the Evaluation of Individual Quality of Life or SEI-QoL [10, 11] was developed to capture the idiosyncratic nature of an individual's experience of well-being. It was developed such that people were able to first select and define their own dimensions of quality of life and then assign a relative weight to each chosen dimension.

The internal reliability of the SEIQoL has ranged from 0.60 to 0.75 whilst test–retest reliability has been reported as 0.88 over 7–10 days. Finally, the Personal Wellbeing Index [12, 13] assesses with a small number of items, both objective and subjective dimensions of quality of life. Along with a global life satisfaction item, Cummins' [12] scale includes eight questions on standard of living, health, sense of achievement, relationships, safety, place in community, future security and spirituality.

Assessment approaches can measure objective data (“How often do you have trouble sleeping?”), subjective evaluations of objective domains (“How happy are you with your relationships?”) or assessments of subjective processes such as one’s own cognitive or affective state (“Rate how distressed you are at the moment.”), or they can measure several constructs at once to captures the multi-factorial dimension of quality of life. These multiple utility instruments not only exhibit strong psychometric properties [14], but also have the advantage of covering a broad range of constructs.

However, whilst as reported, there exist multiple utility instruments that evaluate both objective and subjective domains as derived from general assessments of quality of life, there remains a lack of instruments derived from issues that both present in clinical practice and have an impact upon quality of life. As such it seemed of use to construct a clinically relevant quality of life scale that captured the range of topics reported in clinical interventions, where there was data that an improvement in them correlated with an increase in quality of life.

There seemed however no utility in replicating research that attempted to capture all aspects of QOL. Hence in response to this, as reported in earlier research [15], a preliminary exploratory search of the literature was limited to studies that included the presence of a significant effect from improving a presenting issue (e.g., communication skills), upon the experience of well-being or perceived quality of life domain (e.g., relationships).

Variables that met the above criteria were divided into objective and subjective areas and the search was limited to ten domains so that the number was sufficiently small to be clinically applicable [16]. The identified variables fell under the following ten provisional groupings: relationships, work, money, health, and leisure (objective domains); mindfulness, self-esteem, life events, mental style and life management (subjective domains).

Whilst it had been reported that each of the above areas contained variables that had a significant impact upon well-being, both the validity of the divisions and the extent of overlap remained unknown. For example, reviews by Diener et al. [17] and Diener and Biswas-Diener [18] support the proposition that the so-called objective domains of relationships, work, money, health, and leisure are independent, however the relationships among the more subjective domains is unclear. Rodríguez et al. [19] attempted to address this by dividing subjective wellbeing into four independent constructs: positive affect, negative affect, overall life satisfaction, and salient domains of life.

Based on the above and the more recent research of Jones and Drummond ‘s [15] summary of current findings on quality of life domains, it was expected that some type of division into subjective (affect and cognition) and objective domains would occur. However, it remained of interest to determine which of the identified subjective domains were independent, and which were related and had common underlying processes that were part of a bigger construct.

Furthermore, by drawing variables from clinical studies that demonstrated that variable’s impact upon quality of life, and testing them in a scale, we could build a better relationship between psychological interventions and quality of life research [20]. This could then benefit therapeutic utility by addressing relevant quality of life factors in both initial diagnostic assessments and evaluation of pre-post intervention effects.

What follows is an outline of the subsequent construction, administration and evaluation of the new multiple utility instrument, the Clinical Quality of Life Scale (CLINQOL), which evaluated clinically relevant subjective and objective domains of perceived quality of life and well-being.

## Method

### Questionnaire design

An initial pool of facets was generated from the previous exploratory search of empirical studies that reported significant correlations between perceived quality of life and well-being [15]. The identified areas are relationships, work, money, health and leisure, mindfulness, self-esteem, resolution of past life events, mental style, and life management. One hundred studies (ten studies across each of the domains), were investigated producing 195 variables or facets with an uneven distribution across the domains.

Multi-dimensional facets were simplified or deleted, so as to not leave ambiguity as to what dimension of quality of life was being described. As the facets were to be converted into questionnaire items, it was critical that the subsequent questions would not lead to confusion as to what aspect of quality of life they were addressing. For reasons of redundancy facets identified as having common mediators (e.g., a sense of control) were also deleted, leaving a total of 187 facets.

These were then operationalized in preparation for item development, with some being transformed into positively and negatively worded definitions to reduce response bias. A Likert scale above four data points was chosen (0–10 in this case) and items were collated into a survey format. In light of the research that quality of life reflects an interaction between a life domain and the importance given to it by the individual [7], a provisional weighting scale was initially constructed with participants asked to rate the life domains in terms of importance. The weighting scale was later discarded as redundant as most respondents rated almost all areas as being either important or very important.

An initial pilot of the 187 item iteration of the scale was conducted with 40 adult participants. The response rate, as calculated by the number of usable responses divided by the total number of potential respondents, was 75% [21]. Their age ranged from 18 to 65 and gender was distributed into 32.5% male and 67.5% female. To strengthen content validity and identify weak items, a reliability analysis using Cronbach's alpha was run to determine internal consistency. Analyses of the items of each of the original ten quality of life categories were carried out with all categories demonstrating an internal consistency ranging from 0.613 to 0.860. Whilst 0.80 or greater is seen to be a very good or ideal level, an accepted rule is that an α of 0.60 or 0.70 is still an acceptable level of reliability [22, 23].

Psychometrically weak items were then deleted based upon poor reliability or three or more weak loadings across their respective categories (Weak =  < 0.40 factor loading). Positions vary on cut off values with 0.40 seen by some as an acceptable, albeit conservative value [24]. Typically, stricter cut off values generate models that better fit the data, compared to ones generated using more accommodating cut off values [25], however it was argued that 0.40 enabled the inclusion of more items.

The revised scale consisted of 112 items across the ten categories: Mindfulness (10 items), Self-esteem (10 items), Life Events (10 items), Mental Style (11 items), Life Management (15 items), Relationships (10 items), Work (15 items), Money (10 items), Health (10 items) and Leisure (11 items). To meet the criteria for maintaining an international standard for the measurement of life satisfaction [26], and as a minimum sample size of 200 people was recommended by Cummins [27] to meet this standard so that correlation coefficients were sufficiently robust, it was then administered to 210 people. This sample approximates the independent findings of Schönbrodt and Perugini [28], that in typical scenarios, sample sizes stabilise when they approach 250 subjects.

### Procedure

By way of summary, there were three separate recruitment cycles for each stage of the scale development. The initial 187 item draft of the Clinical Quality of Life Scale (CLINQOL) was piloted on 40 adult participants. The resultant 112 item scale was administered to 210 adult participants, and in a follow up study, the final 54 item scale was evaluated for test–retest reliability, with 127 respondents completing the baseline survey and 106 completing the repeat survey. In each case participants came from separate and unrelated groups from the general population. They all provided informed consent (*study approved by Murdoch University Human Research Ethics Committee*).

The primary recruitment process for the scales was an online university portal that provided access to a pool of volunteers available for research projects. People were able to login, read information about the project, and a general outline of what they were expected to do. Because the portal was a secure site, personal details of volunteers remained confidential. A second recruitment method was chain referral or snowball sampling. This sampling technique was chosen as it is simple, cost-efficient, needs less planning and fewer workforces compared to other sampling techniques. These benefits were seen to outweigh any disadvantages such as less control over the sampling method and its representativeness.

Questionnaire packs were handed out to people within several organisations with diverse occupational roles, thus developing a snowball sample. Each group provided a secure place for the completed questionnaires to be returned in sealed envelopes. The questionnaire in all groups was preceded by an introduction letter, university pro forma ethics and consent forms, and was described as taking around 30 min to complete with the option to receive a general summary of findings.

Once developed a final version of the scale was administered to adult participants who were recruited from a newly developed intervention, the Mindfulness-based Quality of Life and Well-being Program (copy available upon request). Program participants were self-selecting adult volunteers from the English speaking population. This program was divided into three sections: (a) mindfulness theory and methods; (b) application of mindfulness and goal setting to five major life areas (relationships, work, money, health and leisure); (c) application of mindfulness to mental state and well-being. The 2-day version of the program, aggregating 12 h of total content across the three sections, was evaluated using the new Clinical Quality of Life Scale (CLINQOL) and five other standardized measures (see below).

### Scales

To establish convergent validity and compare the scale against already established measures, the Pearson Product Moment Correlation was used to evaluate the strength and direction of the linear relationship between factor scores and related standard measures. The Mindfulness Attention Awareness Scale (MAAS), [29] and the Satisfaction with Life scale (SWL), [6] were chosen to represent the cognitive component of well-being.

The MAAS is a 15-item, self-report measure which correlates with other psychometric measures of mindfulness [30] and has high internal consistency reliability (Cronbach’s alpha = 0.82) [29]. The Satisfaction with Life scale (SWL) has been shown to identify changes in a single factor seen as life satisfaction [31] and has five questions rated from (1) “Strongly disagree” to (7) “Strongly agree”. Diener et al. [6] have reported a Cronbach’s alpha of 0.87 and test–retest reliability of 0.82 at two-month retest.

The Positive and Negative Affect Scale (PANAS) [32] was chosen for its assessment of the affect component of well-being. The 20-item self-report instrument has good internal consistency with a Cronbach’s alpha of 0.88 for positive affect and 0.87 for negative affect and is robust across a range of demographics [33, 34].

The reverse scored Assessment of Quality of Life Instrument (AQoL-8D), [35] and the Personal Wellbeing Index–Adult (PWI-A), [13] were chosen for their coverage of items representative of the 5 objective life domains. The AQoL assesses a broad range of psychosocial aspects of quality of life. Correlations between the AQoL and five other multi-attribute utility instruments averaged 0.69 and its Cronbach alpha coefficient was 0.96 [35]. The PWI-A correlates 0.78 with the Satisfaction with Life scale [36] and Cronbach’s alpha falls between 0.70 and 0.85 with a test–retest reliability correlation coefficient of 0.84 across a 1–2 week interval [13, 37].

### Construct validity

In line with recommendations [38] that factor analysis requires a minimum of five subjects per factor with sample sizes preferably greater than 200, the revised 112 item CLINQOL was administered to 210 adult participants from the general population, with a response rate of 94%. Age ranged from 18 to 80 years with a mean of 46.4 years. Gender was distributed into 40.9% male and 59.1% female with 89.5% employed and 9% retired and 1.5% unemployed. Relationship status included 48.5% married, 20.3% in a relationship and 31.2% single; and the range of children was 0 to 8 with a mean of 1.4.

Assumptions of normality were met with the Kolmogorov–Smirnov statistic producing a Lilliefors Significance level of greater than 0.05 (0.052), as were assumptions of linearity (normal probability plot cases falling more or less in a straight line). The impact of outliers was investigated with the deletion of the top and bottom 5% of the data revealing a marginal difference in mean scores (Mean = 70.00, 5% Trim Mean = 70.40) allowing the 210 scores to be kept for analysis. Bartlett's test of sphericity, to measure that variables are related and suitable for structure detection, was large and significant (*p* < 0.001), and the Kaiser–Meyer–Olkin measure of sampling adequacy was greater than the suggested minimum of 0.6 (0.795), giving further evidence of factorability. The internal consistency of the interim scale using Cronbach’s coefficient alpha was 0.958, suggesting good internal consistency.

Inspection of an initial correlation matrix of the 112 item scale indicated a sufficiently low level of multi-collinearity suitable for factoring [39]. As the aim was to create fewer index variables from a larger set of measured variables, the unidimensionality and distinctiveness of the ten categories was investigated with Principal Components Analysis (PCA) [40, 41].

A PCA was chosen based on the assumption that, as all items were identified as correlated with quality of life, they would correlate with each other and hence share common variance. Because the goal was to explain as much of the total variance in the variables as possible, and to identify if the proposed life domains were independent, a principal components analysis was used to reduce the data into a smaller number of components [42, 43] (SPSS Statistics also uses PCA as its default algorithm for conducting factor analysis). On reflection whilst quality of life may have made up the common variance for all items, it could also be argued that within each item there may also have been some specific and error variance, such as systematic factors like affect or optimism, which would justify the choice of an exploratory factor analysis.

A varimax rotation was chosen both because the 112 items were not designed to measure single or related constructs but rather orthogonal factors, and because the rotated solution is easier to interpret than oblique rotation [44, 45]. The initial rotation converged in 23 iterations and was evaluated for possible factor clustering and multiple item loadings. Psychometrically weak items were again deleted based upon poor reliability (Weak =  < 0.40 factor loading) or multiple lower loading across factors, leaving a final 54 item scale. A varimax rotation was again performed on the remaining items that represented the ten originally identified areas, yielding seven clearly defined factors emerging in 7 iterations (Table 1).

**Table 1:** Varimax Rotation of 112 item scale

Item		Greatest Beta
**Factor 1: Mental Style**		
96	I tend to get stuck in the negative emotions of past events	− .75
57	I tend to interpret past events in a negative way	− .72
99	I find it hard to emotionally disengage from stressful events	− .72
32	I frequently dwell on past negative events	− .69
12	I focus or dwell on the details of past negative events	− .65
1	I can easily stop negative thoughts	.62
105	I worry about things more than I should	− .61
107	I am able to let go of the negative effect of upsetting events	.58
11	I’m good at managing stress	.57
27	I have more a tense than relaxed response to everyday events	− .56
77	I find it hard to adjust to changes to my routine	− .55
40	I find it hard to relax when I take breaks	− .52
74	I look for the positive in things	.49
66	I get overwhelmed during demanding times	− .48
29	I can deal with and express emotions at the right time and place	.48
89	I feel unconfident about my ability to cope with stressful events	− .43
79	I find it hard to stay focused in the present moment	− .40
109	I have a sense of self-worth that is independent of others	.40
**Factor 2: Life Management**		
50	I’m quite effective and successful in the tasks I set myself	.71
8	I always make progress in the goals I set myself	.66
28	I set goals for my life	.62
49	If I have a big project I break it up into small tasks	.59
31	I set priorities in my life which I live by	.58
59	I’ve got good focus when it comes to completing tasks	.58
70	I often don’t finish tasks	− .56
35	My approach to achieving my goals is quite disorganised	− 54
2	I set tasks that I am capable of completing	.47
**Factor 3: Money**		
76	I tend to impulse buy and not manage my money	− .78
68	I consistently spend what I earn without saving anything	− .75
14	I’m undisciplined when it comes to spending money	− .74
94	I often fail to follow a budget	− .70
69	I am careless about paying off debt	− .58
88	I can’t save much money with what I earn	− .45
**Factor 4: Work**		
110	I am bored by the lack of variety in my work	− .71
43	I’m unmotivated in my job	− .66
6	My work offers me little opportunity to develop myself	− .66
90	I feel unsupported in the work I do	− .63
15	I find my work gives me a chance to express who I am	.60
58	I feel safe in my work environment	.60
9	My mood is not that good when I’m at work	− .52
**Factor 5: Health**		
95	I see myself as being in good health	.71
80	I frequently suffer from pain or bodily discomfort	− 66
108	I am able to perform a range of physically demanding tasks	.59
92	I have a high level of daily energy	.54
19	I am as fit as I want to be	.50
82	I suffer from sleep disturbance	− .47
**Factor 6: Leisure**		
78	I am committed to the leisure activities I choose	.67
98	I can get very absorbed in a leisure activity and forget myself	.61
37	I have few hobbies	− .58
41	I am unhappy with the amount of leisure time I have	− .57
64	I use my spare time in a personally fulfilling way	.51
**Factor 7: Relationships**		
100	I feel I understand the personal distress of others	.74
104	I tend to lack sympathy for people sometimes	− .68
51	I’m good at supporting others in emotional or practical ways	.63

Items from the first four so called subjective categories (mindfulness, self-esteem, resolution of the past and mental style) clustered together as one composite subjective factor that was subsequently described as Mental Style. The fifth subjective category of life management separated out as an independent factor and the five objective categories of work, money, health, leisure and relationships also emerged as distinct factors.

The Mental Style factor included three Mindfulness items, one Self-Esteem item, eight Life Events Resolution items and six Mental Style items. Factor two (Life Management) was made up of items centred on planning, goals, and progress such as “I’m quite effective and successful in the tasks I set myself” (0.714), and “I always make progress in the goals I set myself” (0.662). The third factor (Money) included themes of spending discipline, income issues and general money management concerns, with strong items such as “I consistently spend what I earn without saving anything (0.753) and “I often fail to follow a budget” (0.695). Factor four (Work) included issues such as lack of vocational expression, interpersonal conflict and work problems and a sense of well-being about one's work. Strongly loading items included “I am bored by the lack of variety in my work (0.701), “I’m unmotivated in my job” (0.657) and “I find my work gives me a chance to express who I am” (0.601).

The fifth factor (Health) included general health topics, physical well-being and energy and physical problems. Items included “I see myself in good health” (0.709), “I have a high level of daily energy” (0.544), “I frequently suffer from pain or bodily discomfort (0.658), and “I suffer from sleep disturbance” (0.464). The sixth factor (Leisure) included items focusing on personal fulfilment and the experience of stimulation and challenge. Some of these items were “I can get very absorbed in a leisure activity and forget myself” (0.611) and “I am unhappy about the amount of leisure time I have” (0.571). Factor seven was made up of relationship items focusing on empathic, supportive, personal sharing dimensions and items included “I feel I understand the personal distress of others (0.734) and “I’m good at supporting others in emotional or practical ways” (0.625).

### Reliability

Temporal stability along with sensitivity to change is critical for measures of well-being as they must be robust enough to withstand momentary fluctuations in mood states and yet reflect genuine changes in subjective experience [46]. As reliability estimates are used to evaluate the stability of measures administered at different times to the same individuals, in a follow up study the CLINQOL was evaluated for its test–retest reliability [47].

A total of 127 respondents completed a baseline survey and 106 completed the second-stage survey 14 days later, with a response rate of 79%. Participants were recruited from the Mindfulness-based Quality of Life and Well-being Program in which they were participating. A skewness analysis revealed all variables to be normally distributed, however six outliers were detected and deleted, leaving a sample of 100 subjects. Ages ranged from 18 to 77 years with a mean of 42.1 years, gender was distributed into 12% male and 88% female, with 88.2% employed, 6.5% retired and 5.2% unemployed. Relationship status included 50.65% married, 20.5% in a relationship and 28.9% single; and the range of children was 0 to 5 with a mean of 1.3.

To evaluate temporal stability, a reliability analysis using Pearson’s r correlation was run. For group data, a correlation of at least 0.7 is recommended as evidence of satisfactory reliability, with coefficients of 0.9 considered acceptable at the individual level for clinical purposes [35]. The CLINQOL showed strong temporal stability at a group level and was close to the clinical threshold (Pearson’s r = 0.85, n = 100), suggesting the index has moderate to strong test–retest reliability across a 2 week interval.

The seven factors (Mental Style, Life Management, Relationships, Work, Money, Health, and Leisure) were also analysed to identify the test–retest stability of each category (Table 2). A skewness analysis again revealed all variables to be normally distributed with no significant outliers. Mental Style, Money and Health demonstrated satisfactory reliability, whilst Life Management, Relationships, Work, and Leisure failed to maintain stability over time, suggesting that the instrument should be used as a composite only.

**Table 2:** CLINQOL Factors Test–Retest Reliability Means, S.D.s and coefficients (n = 100)

CLINQOL	Baseline		2-weeks		
Dimensions	M	*SD*	*M*	*SD*	*r*
CLINQOL	288.56	60.42	295.58	62.44	.85
Mental Style	84.24	27.51	89.53	29.40	.77
Life Management	50.82	13.50	49.17	17.47	.60
Relationships	21.05	5.84	19.68	6.88	.60
Work	39.91	11.46	42.55	12.08	.52
Money	39.30	12.47	42.20	15.24	.72
Health	26.6	11.46	27.02	12.40	.82
Leisure	25.62	7.07	25.38	9.54	.47

### Concurrent validity

As the key indicators of the quality of a measuring instrument are the reliability and validity of the measures [48], the new 54 item Clinical Quality of Life Scale (CLINQOL) was administered to an additional 59 people to assess its concurrent validity (see Additional file 1: Appendix A). Participants were again recruited from the same Mindfulness-based Quality of Life and Well-being Program in which they were participating. They had an age range of 23–74 years with a median age of 45.7 years. The gender ratio was 86% females and 14% males with 96.5% employed and 3.5% unemployed. Relationship status included 45.7% married, 25.4% in a relationship and 28.9% single; and the range of children was 0 to 4 with a mean of 1.4.

The seven factors (Mental Style, Life Management, Relationships, Work, Money, Health, and Leisure) were analysed to establish means, standard deviations, and level of internal consistency. To evaluate internal consistency, a reliability analysis using Cronbach’s alpha was run for each category (Table 3). Prior to conducting the analyses, a skewness analysis revealed all variables to be normally distributed with no significant outliers detected upon application of the outlier labelling rule [49].

**Table 3:** CLINQOL (54 Items) Means, S.D.s and alpha coefficients

QOL Domain	Items	*M*	*SD*	*Alpha*
Mental Style	18	4.74	1.38	.909
Life Management	9	6.11	1.56	.848
Relationships	3	6.07	1.03	.504
Work	7	6.08	1.48	.822
Money	6	6.19	2.20	.867
Health	6	5.31	1.74	.648
Leisure	5	5.34	1.74	.702

Whilst the intention of the CLINQOL scale was to primarily generate an overall scale score, it was also of clinical interest to generate individual sub-scale scores. Items within each subscale were evaluated with Cronbach’s coefficient alpha ranging from 0.504 to 0.904. All items were also subjected to a reliability test of the unidimensional construct of quality of life measured by the CLINQOL, and the overall reliability of the total scale was 0.907 which is above the acceptable minimum of 0.70 for a scale that is newly developed [50, 51].

Pearson’s r correlation revealed that there were significant relationships between the CLINQOL and all other related scales, suggesting that it demonstrated sufficient concurrent validity to be a suitable instrument to assess self-reported perception of quality of life and subjective well-being (Table 3). A correlation analysis was conducted to investigate relationships between the individual CLINQOL factors and each scale. There were significant relationships between Mental Style, Work, Health and Leisure with all measures, and Life Management correlated with all other scales except negative affect (PANAS-NA). Money correlated only with quality of life (AqoL) and subjective well-being (PWI), and Relationships did not correlate with any measure (Table 4).

**Table 4:** Correlational matrix for CLINQOL (total score and factors) and dependent variables (n = 59)

	MAAS	AqoL	PWI	PA	NA	SWLS
CLINQOL score	.557**	− .739**	.686**	.573**	− .571**	.590**
Mental Style	.488**	− .597**	.544**	.429**	− .433**	.479**
Life Management	.343**	− .351**	.371**	.300*	− .200	.266*
Relationships	− .037	− .004	.017	.007	− .158	− .145
Work	.265*	− .468**	.615**	.510**	− .493**	.521**
Money	.228	− .327*	.274*	.157	− .119	.176
Health	.329*	− .642**	.493**	.385**	− .335**	.487**
Leisure	.370**	− .359**	.390**	.392**	− .229**	.388**

For mindfulness (MAAS), quality of life (AqoL), subjective well-being (PWI), positive and negative affect (PANAS) and life satisfaction (SWLS) (*p < 0.05, **p < 0.01)

The Clinical Quality of Life Scale was also used to evaluate the new mindfulness program, the Mindfulness-based Quality of Life and Well-being Program. The program introduced participants to general mindfulness theory and principles before teaching techniques and exercises. The program was administered and evaluated against the CLINQOL and the previously mentioned measures: Mindfulness Attention Awareness Scale, Quality of Life Index, Personal Wellbeing Index–Adult, Positive and Negative Affect Scale and the Satisfaction with Life scale. Pre-post change scores correlated with both subjective well-being and positive affect but not with the remaining variables (Table 5).

**Table 5:** Pre-post change scores correlation matrix of the 2-day mindfulness program (n = 42)

	CLINQOL	MAAS	AQOL	PWI	PA	NA	SWLS
CLINQOL	1						
MAAS	.031	1					
AqoL	− .281	− .537**	1				
PWI	.313*	.338*	.578**	1			
PA	.361*	.121	− .562**	.393**	1		
NA	− .026	− .349*	.620**	− .443**	− .222	1	
SWLS	.274	.229	− .590**	.531**	.460**	− .353*	1

For the new Clinical Quality of Life Scale (CLINQOL) and mindfulness (MAAS), quality of life (AqoL), subjective well-being (PWI), positive and negative affect (PANAS) and life satisfaction (SWLS) (*p < 0.05, **p < 0.01)

It was speculated whether the negative and positive items from the Mental Style sub-domain of the CLINQOL (e.g., “I tend to interpret past events in a negative way” or “I look for the positive in things”) would correlate with the respective dimensions of affect in the Positive and Negative Affect Scale. This was supported with eight of the twelve negatively phrased items correlating with negative affect, and three of the six positive items correlating with positive affect (Table 6).

**Table 6:** Correlational matrix for negative and positive Mental Style items with negative and positive affect (n = 59) (*p < 0.05, **p < *0.01)*

Item		Positive affect	Negative affect
1	I can easily stop negative thoughts	.149	
2	I find it hard to stay focused in the present moment		− .011
3	I have more a tense than relaxed response to everyday events		.412**
4	I have a sense of self-worth that is independent of others	.203	
5	I focus or dwell on the details of past negative events		.116
6	I tend to get stuck in the negative emotions of past events		.311*
7	I am able to let go of the negative effect of upsetting events	.269*	
8	I tend to interpret past events in a negative way		.309*
9	I look for the positive in things	.402**	
10	I get overwhelmed during demanding times		.318*
11	I frequently dwell on past negative events		.433**
12	I feel unconfident about my ability to cope with stressful events		.336*
13	I worry about things more than I should		.321*
14	I’m good at managing stress	.447**	
15	I find it hard to adjust to changes to my routine		.170
16	I find it hard to emotionally disengage from stressful events		.235
17	I find it hard to relax when I take breaks		.355**
18	I can deal with and express emotions at the right time and place	.075	

## Discussion

The purpose of this study was to develop and validate a new quality of life and well-being measure that had clinical application. This was in response to an exploratory examination of the literature that revealed that there was a scarcity of clinically useful scales that assessed both objective life areas (relationships) and subjective areas (mental control) in one scale. A reliability analysis of the newly constructed Clinical Quality of Life Scale found both that the 54-item measure formed internally consistent scales and that the scale demonstrated concurrent validity with all measures. The principal components analysis identified seven clearly defined factors. Items from the first four subjective domains clustered as one composite factor subsequently labelled as Mental Style with the fifth subjective domain of life management emerging as a separate factor. The remaining five objective domains of work, money, health, leisure, and relationships all formed distinct factors.

The first factor of Mental Style, included the four proposed groupings of mindfulness, self-esteem, life events resolution and mental style, that were found to be correlated with well-being [52–55]. One could ask why, when their facets, derived from the clinical literature that represented these domains, were itemized in a questionnaire format, that they did not separate out as independent factors, but rather clustered together under one general factor.

One explanation is that items within this factor such as “I’m good at managing stress” and “I find it hard to relax when I take breaks” may be play a buffering role and converge on general coping behaviours that make up resilience [56], or the ability to maintain or regain psychological well-being and homeostasis [57]. Whilst mental style and stress management may not appear seem to share functional homogeneity, in their classifications of the different forms of coping, Skinner et al. [41] indicate that resilience is a component of mental style.

There are some indications that the composite Mental Style factor may have captured pivotal aspects of well-being. For example, it moderately correlated with all measures of quality of life, subjective well-being, life satisfaction, and both positive and negative affect (*p* < 0.01). In regards of affect, two thirds of the negatively phrased Mental Style items correlated with negative affect, and half of the positive items correlated with positive affect. It would be of interest to explore, in view of items being initially generated from the areas of mindfulness, self-esteem, life events resolution and mental style, if the subscale may have sufficient breadth to operate as a short-form to assess these components of well-being.

It was unexpected that the second factor of Life Management was the only category of the five subjective groupings to emerge as a factor. This lends some support to the research that items centring on the themes of planning, goals and progress share a functional homogeneity. This is in line with the three dimensions that Brunstein [58] found mediated the effects of goal achievement upon subjective well-being: the degree of commitment to one’s goals, the attainability of the set goal states and progress towards set goals [59].

The generation of this factor is supported by the findings from motivational theory [60, 61] that goal setting [62, 63], has a strong relationship to subjective well-being [64, 65]. This suggests that having a well organised, skilful, and balanced lifestyle [66], and the perception of achievement of personal goals, is associated with improvement in emotion regulation [67], and increased well-being [68]. Perhaps this factor bridges the subjective and objective domains and represents the mental processes required to successfully manage life areas.

The remaining five factors generated by the principal components analysis corresponded with the proposed five objective domains of money, work, health, leisure and relationships. The factor of Money centred on themes of spending discipline, income issues and general money management concerns. This is in line with research that budgeting [69], relative income [70, 71] and money management attitudes [72, 73] play an important role in people’s relationship to money.

The factor of Work included well documented issues of vocational expression [74, 75], conflict and work problems [76, 77] and a sense of well-being about one’s work [78–80]. Similarly, the factor of Health was a mixture of items related to general health topics (“I am able to perform a range of physically demanding tasks”), physical problems (“I frequently suffer from pain or bodily discomfort”, “I suffer from sleep disturbance”) and physical well-being and energy (“I see myself in good health”, “I have a high level of daily energy”). Such diversity would suggest a clear multi-factorial picture of health rather than a functionally homogeneous one, a picture which aligns with the literature [81, 82, 84, 93].

The factor of Leisure included general leisure items, leisure habits, the dimension of personal fulfilment and the experience of stimulation and challenge. This amalgam is in line with factor analytic studies that have identified clusters such as novelty, relaxation, creative expression and cognitive stimulation [85], achievement leisure which can often be competitive and personally challenging [86], and fulfilment or flow activities [87, 88]. This mix is also in line with leisure taxonomies [89] and evidence of the multi-factorial nature of the leisure construct [90, 91].

The final factor was made up of Relationship items focusing on empathy (“I feel I understand the personal distress of others”) and social support items (“I’m good at supporting others in emotional or practical ways”). This is consistent with relationship satisfaction research that has found empathic concern [92], perspective taking [93] and the ability to feel the personal distress of others as predictive of partner satisfaction and well-being [94–96].

It is of interest that five of the seven CLINQOL subscales (Mental state, Life management, Work, Health and Leisure) correlated with all the five standard measures of mindfulness (MAAS), life satisfaction (SWL), positive and negative affect (PANAS), quality of life (AqoL-8D), and well-being (PWI-A), but the 6-item subscale of Money (alpha = 0.867) was associated with quality of life and subjective well-being but not mindfulness, affect or life satisfaction. This mixed finding is consistent with the research that money has a complex relationship with well-being [97]. For example, whilst money can be positively related to well-being, that effect can be reversed if material goals are prized more than other values or if people are struggling financially [18, 98].

### Limitations and recommendations

Whilst the ten domains chosen were identified in a preliminary exploratory search of the literature, recommendations for future research could include a systematic review that applied the same clinical focus. This would reduce selection bias and ensure a more comprehensive picture of clinically relevant quality of life factors, and as a result possibly further domains be included in the subsequent analysis. For example, in the domain of relationships more fine-grained analysis could be achieved if this was separated into extra sub-categories such as family, friends and intimate relationships.

There were several weaknesses in the scale construction that could be improved in a further iteration. Firstly, due to low factor loadings, the original ten items in the Relationships category were reduced to three in the final scale. The subsequent psychometric weakness of this domain could explain why, unlike the other subscales, it did not correlate with the standardized measures. Whilst the primary structural model underpinning the CLINQOL is a unidimensional model with items loading on a single global factor, the subscales still provide useful data on each of the life domains. As such data from this domain could be clinically useful to flag relationship issues, and this deficit could be addressed in a revised scale by revisiting the original 10 relationship items to see whether more items could be retained.

In terms of the choice of factor rotation, of the two methods, orthogonal or oblique rotation, the latter is typically employed when factors are not correlated, whilst oblique rotation is used when the obtained factors are related [99]. As the data revealed that the factors were indeed related it is reasonable to argue that obliminal or similar rotations should be performed as it can allow for the best fit of the model to the data that has been gathered [100].

The choice of varimax however still has some justification as clarified by Kim and Mueller [45], “Even the issue of whether factors are correlated or not may not make much difference in the exploratory stages of analysis. It even can be argued that employing a method of orthogonal rotation (or maintaining the arbitrary imposition that the factors remain orthogonal) may be preferred over oblique rotation, if for no other reason than that the former is much simpler to understand and interpret.” (p. 50). They go on to highlight that “If identification of the basic structuring of variables into theoretically meaningful subdimensions is the primary concern of the researcher, as is often the case in an exploratory factor analysis, almost any readily available method of rotation will do the job.” As this was the primary goal of the research there seemed some theoretical precedent to stay with a varimax rotation.

It is recommended that in future research, as an exploratory factor analysis (EFA) has been conducted, a confirmatory factor analysis (CFA) using an independent sample would be of value to provide further data to verify the factor structure of the seven correlated factors. The rationale for this is that if one’s model uses items or constructs that haven’t been tested before for reliability and validity, it is recommended to start with an EFA followed by CFA [40]. It is argued that in the case of a newly developed questionnaire, like the CLINQOL, EFA is recommended to factorize and construct the model from a large data set, however in the case of a revised or adapted questionnaire CFA should be applied.

Furthermore, while its possible to do an EFA and a CFA as a split-sample cross-validation if your sample is large enough, it is also recommended to use different samples of the same target population [101]. That is EFA can be conducted to extract factors from a pilot dataset for the first time, followed by a CFA to validate the factors extracted from an independent dataset. This way the EFA can identify the dimensionality of items from the initial pilot study data and drop items with low factor loadings and redundant items. Once obtained one can proceed to a CFA to assess the unidimensionality, validity, and reliability of the constructs and confirm the model.

Secondly, during the initial scale construction, the attempt to retain only items with high internal consistency resulted in a skewed distribution of items across the seven CLINQOL subscales. For example, the Mental State subscale, due to it being a conflation of the four original subjective domains ended up having significantly more items than the other subscales. In a further revision, in contrast to increasing the number of usable Relationship items, a reduction of the number of Mental State items is recommended to avoid any redundancy. Furthermore, whilst Cronbach's alpha is the most used method to estimate internal consistency [102], in view of the research that it can lead to an overestimation of reliability [103], the omega coefficient [104] may have provided more precise estimates.

When the original 195 variables were transformed into items, they were evenly phrased in either negative or positive terms to avoid response bias. However, when psychometrically weak items were deleted based upon poor reliability, this spread was lost. In any future iterations, it may be worth exploring the relative merits of optimal reliability versus possible increased response bias. Similarly, in a revised scale increasing the cut off values from 0.40 to 0.50 or 0.60 would reduce the number of items and the size of an already long scale, and in doing so improve the goodness of fit between the data and the proposed model [25].

Gender, whilst fairly evenly distributed in the investigation of the 112 item scale, was more skewed in the sample used to determine the final 54 item scale. Whilst gender differences in quality of life have not been considered or found to be of great significance in early quality of life research [5, 105], subsequent research using more fine sex disaggregated data tools such as the Gender Inequality Index have shown otherwise [106, 107].

For example, whilst women typically report lower subjective well-being than men in many settings, often connected to less opportunity, and though Eckermann [108] found females rate higher in social support, Cummins [109] found that males consistently were overall less satisfied than females. In view of the mixed findings, further evaluations of the scale could have a more even mix of males and females to rule out any possible skewing of the data.

It is of value to have therapeutically relevant psychometric instruments that can gauge the pre-intervention state of the client and measure change. As CLINQOL total scores correlated with all the standardised measures, and correlations for most of its subscales also achieved statistical significance, the CLINQOL may be sufficiently representative. Furthermore, as items represented significant clinical predictors of perceived quality of life, the scale could be a useful multiple utility instrument for clinicians to use therapeutically.

Initially, it could serve as a comprehensive assessment of what objective life areas (e.g., relationships) and subjective areas (e.g., mental control) need attention. The identified areas could either be a therapeutic focal point or included as part of a wider multi-dimensional intervention, and then re-assessed post treatment. This use of the scale could go some way to address the lack of rigour clinicians often have in testing for post-intervention change in well-being.

The development of the new measure attempted to address the lack of clinically useful scales that assess both objective life areas (e.g., relationships), and subjective areas (e.g., mental style), in one scale. As it was derived from an investigation of the quality of life and well-being literature, and covers clinically-relevant domains, the scale may serve as a useful multiple utility instrument to assist a clinician’s ability to assess the efficacy of their interventions to improve client quality of life and well-being.

## Supplementary Information

**Additional file 1**. Clinical Quality of Life Scale (CLINQOL).

## Data Availability

The datasets generated during and/or analysed during the current study are available from the corresponding author on reasonable request.
