# Supplementary figures and images for: The construction of a new Clinical Quality of Life Scale (CLINQOL)
Source: BMC Psychol. 2022 Aug 30;10:210. doi: 10.1186/s40359-022-00912-7 (PMC9429599; doi:10.1186/s40359-022-00912-7)

1. CLINQOL Questionnaire


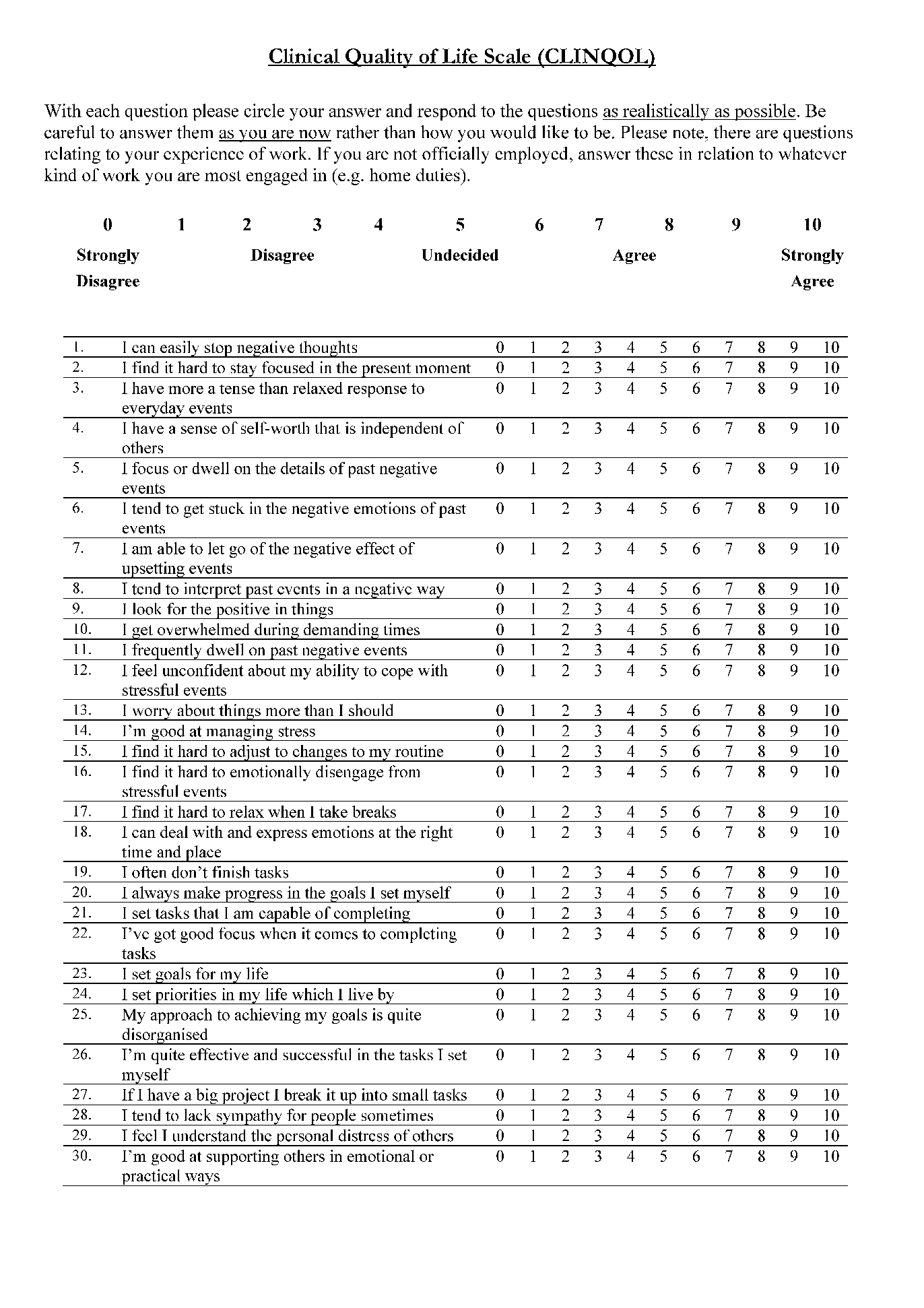


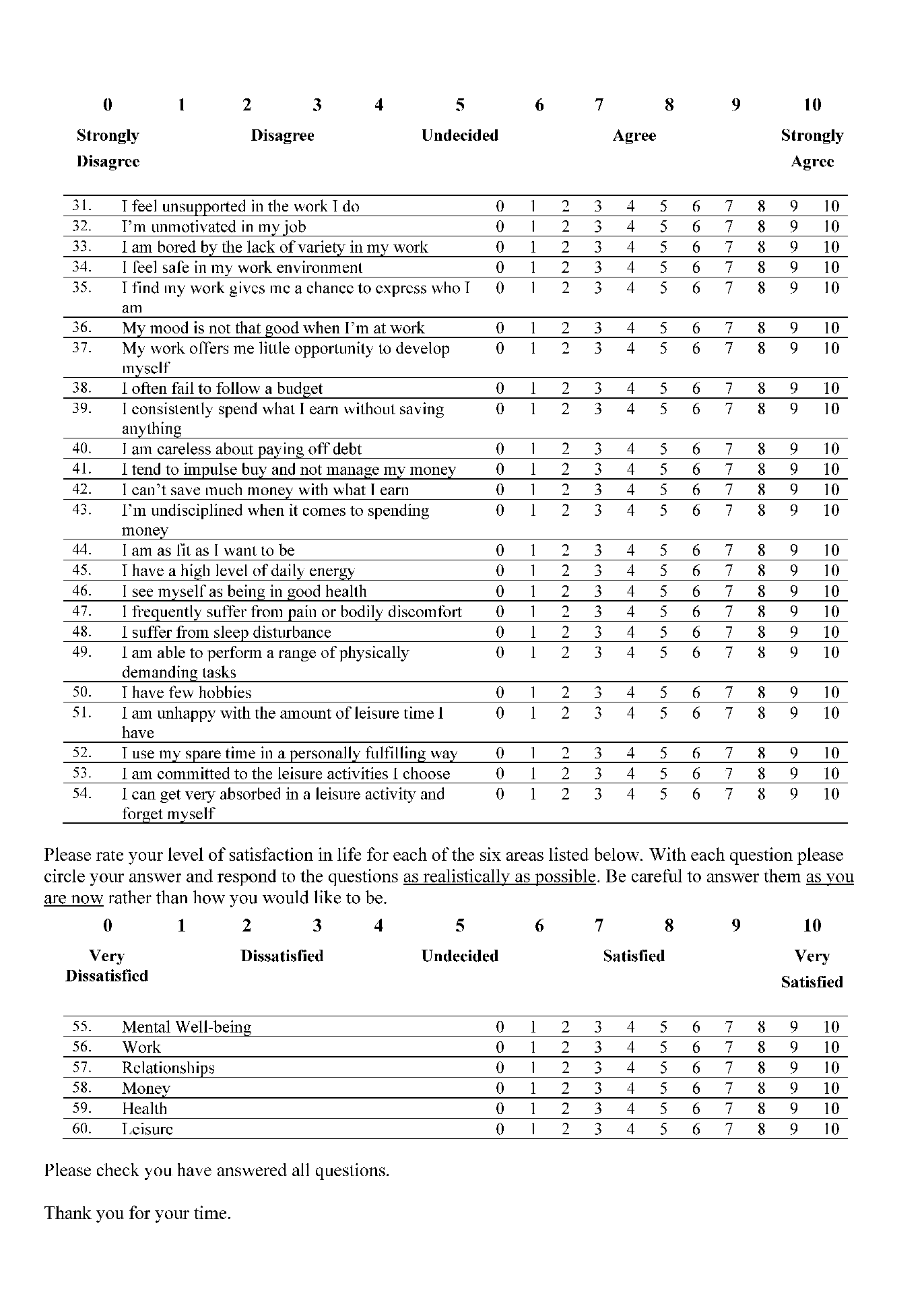

Supplement: Supplementary file 1 — Additional file 1. Clinical Quality of Life Scale (CLINQOL). [file 40359_2022_912_MOESM1_ESM.docx]
